# A Video Teaching Tool Is Effective for Training Residents in Hip Arthroplasty Templating

**DOI:** 10.7759/cureus.35856

**Published:** 2023-03-07

**Authors:** Joseph A Karam, Anthony Tokarski, Carl Deirmengian, Hope Thalody, Stephanie A Kwan, Joseph Mccahon, Rex Lutz, Paul M Courtney, Gregory K Deirmengian

**Affiliations:** 1 Orthopaedic Surgery, University of Illinois at Chicago, Chicago, USA; 2 Orthopaedic Surgery, Rothman Orthopaedic Institute, Philadelphia, USA; 3 Orthopaedic Surgery, Jefferson Health New Jersey, Stratford, USA

**Keywords:** orthopedic surgery, total joint replacement, primary total hip arthroplasty, preoperative templating, medical resident education

## Abstract

Work hour restrictions imposed on orthopedic surgery residents since the early 2000s have reduced educational opportunities at the workplace and encouraged alternative strategies for teaching outside the clinical setting. Preoperative templating is essential for safe and effective total hip arthroplasty (THA) and is accurate in predicting final implants. We sought to determine the effectiveness of a video tool for teaching orthopedic residents basic THA templating skills.

We developed a video-based teaching tool with instructions on proper THA templating techniques. Ten cases were selected for testing, after excluding patients with severe hip deformities and poor-quality radiographs and only retaining those with concordance between templating by the senior authors and implanted components. The study subjects included three postgraduate year 1 (PGY-1), three PGY-2, and three PGY-5 residents, and three adult reconstruction fellows (PGY-6). Templating skills were assessed before and after watching the instructional video. The evaluation included the size and positioning of femoral and acetabular components, as well as the restoration of leg length. Each templating session was repeated twice. Variance was measured to evaluate consistency in measurements. A linear mixed model and F-test were used for statistical analyses.

The number of years in training significantly affected performance prior to exposure to the instructional video. Post-exposure, there was a significant improvement in the accuracy of sizing and positioning of acetabular and femoral components for PGY-1, PGY-2, and PGY-5 residents. The results achieved were comparable to PGY-6 examiners, who did not gain substantial performance benefits from the instructional video. Limb length restoration was less affected by experience or exposure to the video. Component positioning and sizing, as well as leg length discrepancy (LLD), showed a significant decrease in variance after the intervention in all study groups.

Video learning is reliable in teaching invaluable skills to orthopedic surgery residents without encroaching on work hours. We conceived a concise video to train orthopedic residents to perform THA templating with proper technique and demonstrated its efficiency and reproducibility.

## Introduction

In 2003, the Accreditation Council for Graduate Medical Education (ACGME) set an 80-hour restriction on weekly resident work hours in an effort to minimize fatigue-related medical errors. While the success of this policy in improving patient safety has been a topic of debate, it inherently leads to a reduction in the number of clinical encounters and opportunities for resident education [1-4]. As a result, the 80-hour workweek restriction has forced an effort to develop and evaluate strategies for streamlining resident education. Preoperative planning is an essential skill that is necessary for the safe and effective performance of total hip arthroplasty (THA). As part of the preoperative planning process, radiographs are templated to predict the appropriate component sizes and positions and plan the reconstructive goals of surgery, such as leg length and offset restoration. Digital templating has been shown to successfully predict final components within one size in 78%-95% of cases [5-11]. Radiographic templating is a skill that has also been shown to improve with experience [5,6]. Trainees typically acquire and develop this skill throughout their years of training via didactic lectures, individualized learning, and/or direct clinical experience. As for other important clinical and surgical skills that must be acquired through residency training, the weekly 80-hour work restriction limits such opportunities for learning [3]. The American Board of Orthopaedic Surgery (ABOS) and the Residency Review Committee (RRC) for Orthopaedic Surgery have recently instituted strategies for streamlining resident education, such as the mandate that residency programs institute a surgical skills curriculum [12]. These efforts may substitute for educational time lost due to work hour restrictions. Another approach, which may be used in conjunction, resorts to video teaching tools that promote acquiring necessary skills outside the work setting. As a model of this concept, we sought to determine the effectiveness of a video tool that we developed for teaching THA templating skills [4].

## Materials and methods

This prospective study was approved by the Thomas Jefferson University hospital institutional review board (IRB # 11E.296). The senior corresponding author, who is a board-certified, fellowship-trained adult reconstruction surgeon, developed two video-based teaching tools for THA templating. The first tool involved a four-minute and 27-second video demonstrating the use of the computerized digital templating system (Spectra Picture Archive and Communication System Orthopedic templating software version 5.1; Spectra, Linköping, Sweden). The video provided instruction for the use of the digital templating software, including program access, selection of relevant cases and radiographs, selection of appropriate digital implants, manipulation of the size, offset, positioning, and orientation of the digital implants, and use of length and angle tools. The second teaching tool (Video 1) involved a six-minute and five-second video providing instruction on proper THA templating techniques based on commonly approved templating methods [13,14]. The video demonstrated proper sizing and positioning of components with the goal of restoring the appropriate center of rotation of the hip, femoral and acetabular offset, and equal leg lengths.

**Video 1:** Templating instruction video

For the purpose of this study, the components routinely used by one of the senior authors were selected for digital templating (DePuy Trilock femoral component and Pinnacle acetabular component; DePuy, Raynham, MA, USA). Preoperative and six-week postoperative pelvic radiographic films were reviewed for 29 consecutive cases previously performed by the senior author. Fourteen cases were excluded because they demonstrated inadequate or poor-quality radiographs, severe deformity of the hip, a contralateral THA, or moderate to severe contralateral hip arthritis. Preoperative pelvic radiographs of the remaining 15 cases were templated by the two senior authors using the principles demonstrated in Video 1 while blinded to postoperative X-rays and operative notes. The results were then compared to the actual implant sizes used for reconstruction, as obtained from operative notes. Ten of the 15 cases showed exact concordance between the senior authors’ template results and the actual component sizes. These 10 cases were retained for the study.

Orthopedic surgery trainees who took part in the study included three postgraduate year 1 (PGY-1) residents, three pos-graduate year 2 (PGY-2) residents, three postgraduate year 5 (PGY-5) residents, and three postgraduate year 6 (PGY-6) adult reconstruction fellows. Trainees were chosen among their peers based on their availability during the timeframe of the study. It is important to note that formal didactic training in THA templating was not a part of the resident or fellow teaching curriculum at our institution. All trainees watched the first training video and then placed digital acetabular and femoral component templates for the 10 cases, based on their preexisting skills. The trainees were also instructed to mark the femoral head length of choice on the femoral component. All trainees repeated this task 2-4 weeks later. After another two-week holiday, all trainees watched Video 1 and then templated the 10 cases based on their newly acquired knowledge. All trainees again repeated the task of reviewing Video 1 and then templated the 10 cases 2-4 weeks later. To avoid bias due to recollection of radiographs, the 10 cases were deidentified and organized by patients’ dates of birth for the first two sessions and alphabetically by patients’ last names for the second two sessions.

After each templating session, the results were measured and recorded. These included the implant sizes, the acetabular component inclination (measured as the angle between the inter-teardrop line and the face of the acetabular component), and the varus/valgus angle of the femoral component (as measured by the angle between the anatomical axis of the femur and the long axis of the femoral component). In addition, the vertical distance between the center of the axis of the acetabular component and the chosen neck length of the femoral head was measured. This value was compared with the calculated preoperative leg length discrepancy (LLD).

For each measurement, the error was calculated as the absolute value of the difference between the subjects’ measurements and the correct value. For stem and cup sizes, the correct value was defined as the component sizes used at the time of surgery. For acetabular component inclination, the correct values were defined as a range between 40° and 45°. For femoral component positioning, the correct value was defined as 0°, i.e., the axis of the stem is colinear with the axis of the femur.

A linear mixed model is a statistical model that accounts for fixed and random effects. The model design may be used in cases in which there are repeated measurements on the same statistical unit. A linear mixed model predicting absolute error, treating the 12 subjects (residents/fellows) and the 10 pelvic radiographs as independent random effects, was used to look at the interaction of intervention and year of training while controlling for repeated observations with the same subjects and radiographs. In addition, for each group of subjects within the same PGY level, the standard deviation for the net error (as opposed to the absolute error) was used to determine the variance of pre- and post-intervention measurements. This standard deviation indicates how much variation exists among residents of a given year. The F-test was then used to determine changes in variance after watching the second video. All statistical analyses were conducted using the R statistical platform (R Foundation for Statistical Computing, Vienna, Austria).

## Results

**Figure 1:**
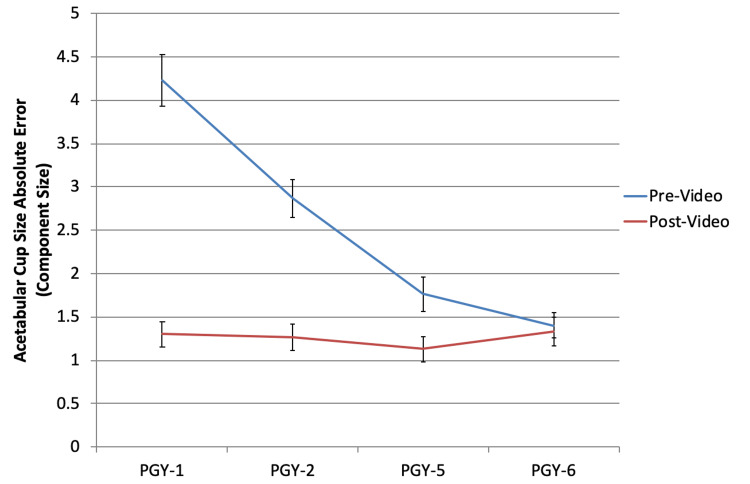
Absolute error in cup size as compared to correct values among the different subject training levels PGY: postgraduate year

**Figure 2:**
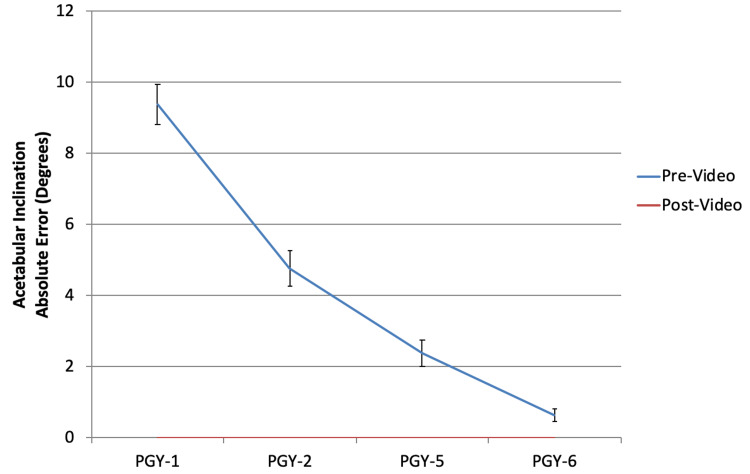
Absolute error in cup inclination in degrees compared to the correct value of 40°-45° among different subject training levels PGY: postgraduate year

**Figure 3:**
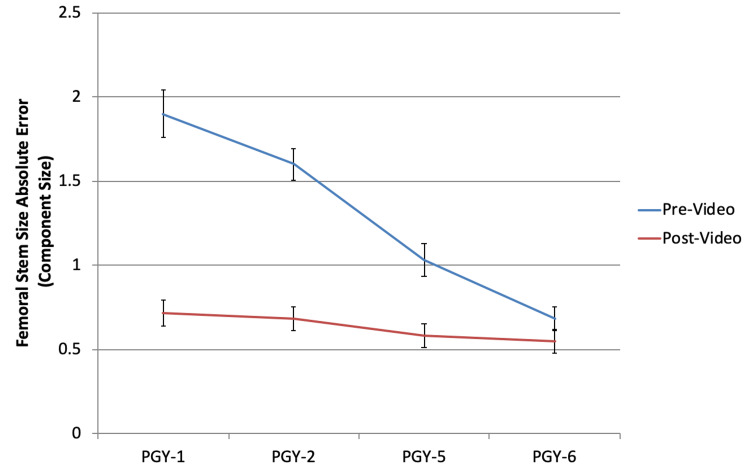
Absolute error in stem size (in number of sizes) as compared to the correct values among the different subject training levels PGY: postgraduate year

**Figure 4:**
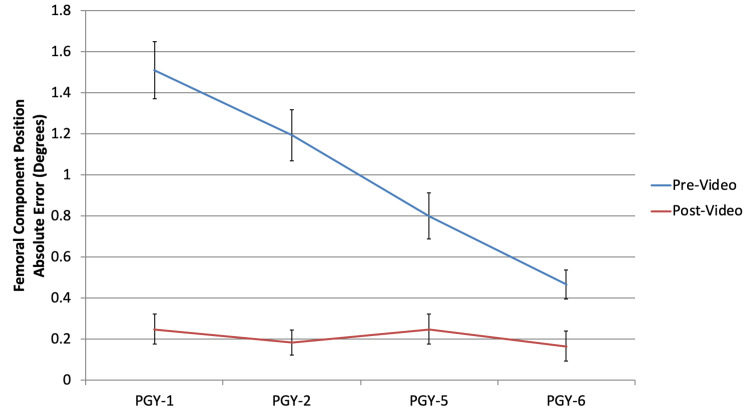
Absolute error in angulation (varus/valgus) of the femoral stem as compared to the correct value among all subject training levels PGY: postgraduate year

**Figure 5:**
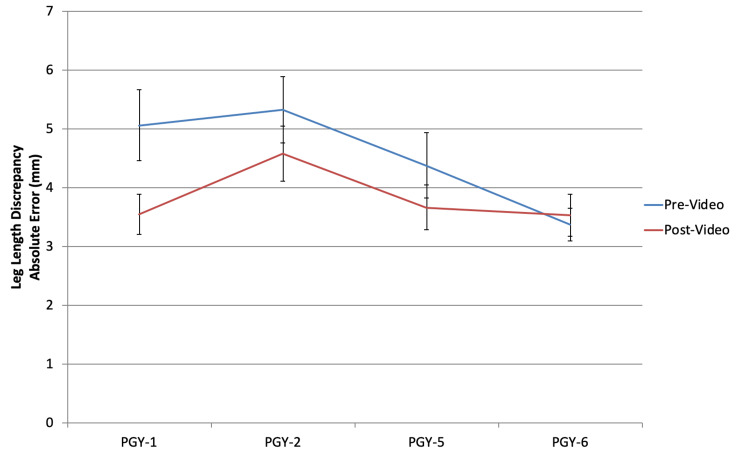
Absolute leg length discrepancy in millimeter based on templating among the different subject training levels PGY: postgraduate year

Acetabular component sizing (Figure 1) and inclination (Figure 2), along with femoral component sizing (Figure 3) and varus/valgus positioning (Figure 4), all showed parallel post-intervention improvement. The net effect of the intervention was that PGY-1, PGY-2, and PGY-5 residents all reached the pre-intervention accuracy of PGY-6 fellows, while the latter showed no statistically significant improvement (p>0.05). The intervention by year interaction was significant in all of these cases (p<0.001). An exception to this trend was leg length restoration (Figure 5), where there was no significant interaction effect (p=0.296), but both year and intervention independently affected absolute measurement error (p=0.023 and p=0.022, respectively).

**Figure 6:**
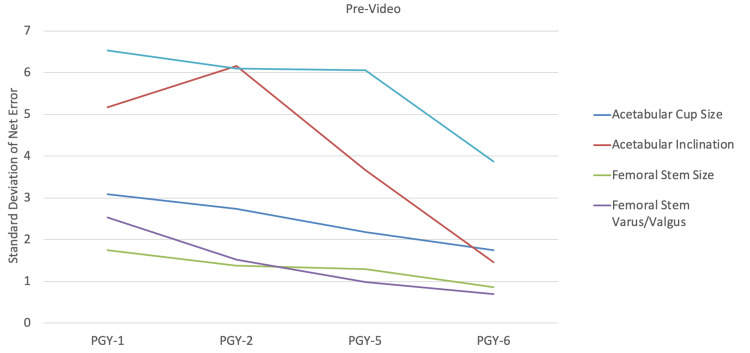
Standard deviation of net error for each measurement by subject training level pre-video PGY: postgraduate year

**Figure 7:**
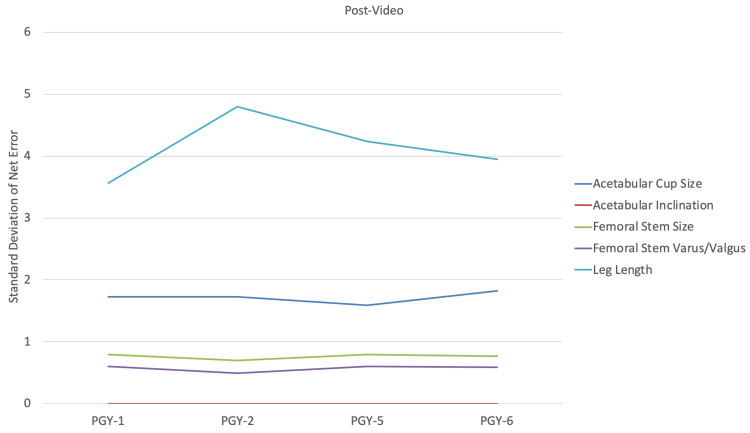
Standard deviation of net error for each measurement by subject training level post-video PGY: postgraduate year

Another improvement was in the variability of estimates. Figure 6 and Figure 7 show that the trend is for the standard deviation post-intervention to fall to the levels of PGY-6 fellows pre-intervention. F-tests for the decline in variance after the intervention are significant for PGY-1, PGY-2, and PGY-5 residents in regard to acetabular component size and inclination, and femoral component size and varus/valgus positioning (p<0.001); they were not significant for PGY-6 fellows (p>0.05). This trend holds true even for leg length, which showed the greatest variation.

## Discussion

The advent of ACGME-mandated restriction of resident work hours has translated into difficulties in effectively training orthopedic surgery residents in a timely manner [2,3]. As a result, it has become necessary to streamline resident education and develop methods of delivering skills outside of the clinical setting. As a model of this concept, we sought to evaluate the effectiveness of a video tool that we developed for teaching residents primary THA digital templating skills.

Video learning is becoming increasingly widespread and has been shown to significantly improve medical student and resident physician knowledge and skills [15,16]. Technical skills such as applying casts or splints and performing arthrocentesis or proper suturing techniques have been shown to be substantially enhanced when adding a video to more traditional methods of learning such as formal lectures or printed sources [17-23]. Mendez et al. used a high-definition instructional video to teach neck dissection and proved its efficiency by testing it on six otolaryngology trainees [24]. As a group, the residents demonstrated decreased surgical errors and attended takeovers when performing neck dissection. Struemph et al. showed that after watching a three-minute instructional video, PGY-1 residents who had limited exposure to bone cement were able to achieve a cement mixing technique comparable to PGY-5 residents who have much more advanced experience [25]. In addition to being highly effective, videos have been shown to be a favored tool for learning in residency. In a survey among general surgery residents about their preferred educational tool to learn a specific cardiothoracic surgery procedure, the majority reported a preference for a short video [26].

Templating in THA helps establish an adequate preoperative plan, identify individual osseous morphology, and prevent complications such as LLD, instability from component malpositioning, implant loosening from undersized cementless implants, and periprosthetic fracture from oversized components [27]. Digital templating is easy to use and has been shown to be very accurate, with several studies demonstrating over 90% ability to predict components within one size of the final implants [7,10,28].

The results of our investigation demonstrated that prior to learning from our video tool, residents of increasing PGY levels showed a corresponding increase in accuracy for the selection of acetabular and femoral component sizes, femoral varus/valgus positioning, and acetabular component inclination. This goes along with prior literature showing increasing templating accuracy with experience [5,6]. Mittag et al. compared templating skills of orthopedic surgery residents to those of an experienced surgeon [5]. In their study, residents predicted the acetabular component in 63% of cases and the femoral component in 89% of cases when compared to final components placed operatively. They significantly underperformed compared to the experienced surgeon, who predicted the final acetabular component in 88% of cases and the femoral component in 97%. They did not, however, differentiate between the level of training of the residents. Carter et al. compared the performance of a junior orthopedic resident, a senior orthopedic resident, and an attending surgeon using conventional templating techniques and also demonstrated improved templating accuracy with experience [6]. Hsu et al. evaluated the THA and knee arthroplasty templating performance of an implant sales representative, a physician assistant, a medical student, an orthopedic surgery resident, and an arthroplasty fellowship-trained orthopedic attending surgeon [29]. They found good interobserver reliability for THA templating among all examiners and excellent intraobserver reliability when looking at the orthopedic surgery resident and the arthroplasty surgeon. They concluded that digital templating could be easily learned and reproduced without advanced orthopedic training.

After watching the instructional video, our study subjects demonstrated improvement in both performance, as represented by means of component positioning and size, as well as consistency, as demonstrated by decreased variance. Junior and senior residents were able to perform at the same level as arthroplasty fellows after watching the video. Adult reconstruction fellows showed to be proficient at properly sizing and positioning components, even without watching the instructional video. In regard to accurate templating of LLD, although post-intervention performance witnessed more consistency, it was still suboptimal.

Our study did present several limitations. We only included simple cases of patients with hip osteoarthritis undergoing THA. Performance would probably have been different if taking into consideration more complicated cases such as patients with proximal femoral deformities, etiologies other than osteoarthritis, and revision THA. Our study is limited to the residents and fellows training at a single institution with its own background in education on THA templating. A single implant was used for homogeneity of the study; however, in clinical practice, a multitude of implants are used, and most surgeons will adapt the implant to the patient’s osseous anatomy, especially in regard to the proximal femur. We did only include 12 subjects in our study; nonetheless, this is higher than prior studies addressing this topic in the literature [5,29]. It is important to note that our study does not imply that the video teaching tool is better than other teaching tools or methods. It simply demonstrates that a video tool is effective in improving resident templating skills, which may be valuable for self-directed learning.

## Conclusions

Video-based learning is a proficient tool to teach skills that need to be acquired during orthopedic residency, including THA templating. We designed a concise video to teach orthopedic surgery residents how to perform adequate templating for THA and demonstrated its effectiveness and reliability. Making this tool universally available will help orthopedic surgery residents learn proper THA templating in a time-efficient manner, without encroaching on their work hours and clinical duties. In the future, we plan to include advanced instructions addressing complex cases such as severe acetabular dysplasia, proximal femoral deformity, and revision THA.
